# Dysbiosis in chronic periodontitis: Key microbial players and interactions with the human host

**DOI:** 10.1038/s41598-017-03804-8

**Published:** 2017-06-16

**Authors:** Zhi-Luo Deng, Szymon P. Szafrański, Michael Jarek, Sabin Bhuju, Irene Wagner-Döbler

**Affiliations:** 1 Research Group Microbial Communication, Department of Molecular Infection Biology, Helmholtz-Centre for Infection Research (HZI), Braunschweig, Germany; 2 Hannover Medical School (MHH), Hannover, Germany; 3 Genome Analytics, Helmholtz Centre for Infection Research, Braunschweig, Germany

## Abstract

Periodontitis is an extremely prevalent disease worldwide and is driven by complex dysbiotic microbiota. Here we analyzed the transcriptional activity of the periodontal pocket microbiota from all domains of life as well as the human host in health and chronic periodontitis. Bacteria showed strong enrichment of 18 KEGG functional modules in chronic periodontitis, including bacterial chemotaxis, flagellar assembly, type III secretion system, type III CRISPR-Cas system, and two component system proteins. Upregulation of these functions was driven by the red-complex pathogens and candidate pathogens, e.g. *Filifactor alocis*, *Prevotella intermedia*, *Fretibacterium fastidiosum* and *Selenomonas sputigena*. Nine virulence factors were strongly up-regulated, among them the arginine deiminase *arcA* from *Porphyromonas gingivalis* and *Mycoplasma arginini*. Viruses and archaea accounted for about 0.1% and 0.22% of total putative mRNA reads, respectively, and a protozoan, *Entamoeba gingivalis*, was highly enriched in periodontitis. Fourteen human transcripts were enriched in periodontitis, including a gene for a ferric iron binding protein, indicating competition with the microbiota for iron, and genes associated with cancer, namely nucleolar phosphoprotein B23, ankyrin-repeat domain 30B-like protein and beta-enolase. The data provide evidence on the level of gene expression *in vivo* for the potentially severe impact of the dysbiotic microbiota on human health.

## Introduction

Dysbiosis of the human microbiome may play a crucial role in a variety of complex diseases, *e*.*g*. type II diabetes, rheumatoid arthritis, allergy, inflammatory bowel disease, periodontitis and even extreme obesity^1–5^. The transition from the healthy state to dysbiosis is thought to be driven by key microbial players in the community. These species can influence the host immune system and worsen the habitat conditions for the commensals which dominate the healthy symbiotic community^6^. Chronic periodontal disease is a prevalent oral disease worldwide. According to a report from CDC (Centers for Disease Control and Prevention) there are 47.2% of adults over 30 years old that have some form of periodontal disease in the USA^7^, and the situation is also serious in other countries.

The oral microbiome is one of the most complex and dynamic microbial communities in the human body, comprising several hundreds of different species of bacteria. Moreover, archaea, protozoa and viruses are present, and millions of different genes are expressed. Dysbiosis of the oral microbiota can interfere with the normal function of the host immune system resulting in enhanced development of periodontitis^8^. Moreover, periodontal disease is associated with many other complex diseases. For instance, it can increase the risk for cardiovascular disease^9^, rheumatoid arthritis^10^ and cancer^11^. The oral cavity is easily accessible and thus an ideal system to study the interaction between host and microbiome.

The periodontal metatranscriptome contains the transcripts of genes from all members of the microbiota, including bacteria, archaea, viruses and phages, protozoa, and fungi, as well as from the human host. It reflects the activities of all of those groups simultaneously without culturing bias. Moreover, the transcriptional activities reflect the interactions between all members of the community and with the human host *in vivo*. Less than a handful of studies on the metatranscriptome of periodontitis are currently available, and each of them shed light on a different aspect of the complex microbiota. Duran-Pinedo and co-workers demonstrated the expression of putative virulence factors in commensal oral microorganisms^12^ and identified GO terms associated with disease progression^13^. Jorth *et al*. compared microbial communities in healthy and diseased periodontal pockets in the same individual^14^ and found that dysbiotic communities were less diverse but more similar among each other than healthy periodontal pocket communities. They identified key players and the metabolic enzymes that they transcribed, and suggested that although the species composition in periodontal pockets varies widely, the metabolic networks activated in disease are conserved^14^.

Archaea comprise a minor component of the oral community^15^. The dominant Archaeon is *Methanobrevibacter oralis* ^16^ which has been categorized as a periodontal pathogen due to its strong association to disease^17^ and pocket depth^18^. Methanogens have been shown to be co-occurring with *Prevotella intermedia*, a fermentative species, possibly due to interspecies hydrogen transfer^18^.

Viruses are the most abundant living entity on the planet. A number of human viruses can be detected in the oral cavity^15^. The salivary virome is dominated by bacteriophages which may act as reservoirs of virulence factors^19^. Interestingly, CRISPRs from healthy individuals cover a wider phylogenetic host spectrum than those of periodontitis patients^20^. Accordingly, a metatranscriptome study showed that more diverse phages were present in periodontally healthy individuals^21^. Subgingival biofilms contain clearly different viromes in health and periodontal disease^22^. Thus, bacteriophages might be important drivers of the community composition of bacteria in the oral cavity, yet their precise role for disease progression is currently not understood^23^.

Very little indeed is known about protozoa in the oral cavity. Two species are commonly observed, *Entamoeba gingivalis* described for the first time^24^ in 1849 and *Trichomonas tenax* ^15^. Although they are strongly correlated to periodontitis^25^ it is now thought that they are not pathogens, but merely commensals feeding on abundant bacteria and debris associated with poor oral hygiene^15^.

We have previously analyzed the shifts in the taxonomic composition of the microbial communities in subjects with chronic periodontal disease^26^. We then analyzed the metatranscriptomes of those samples^27^ and were able to show significant changes in the composition of the active community as well as enrichment of certain COG categories in periodontitis. Gene expression of the commensal *Prevotella nigrescens* shifted towards pathogenicity in samples from individuals with chronic periodontitis, confirming the concept of accessory pathogens. A detailed comparison of butyrate synthesis related transcripts showed that, contrary to previous assumptions, *Fusobacterium nucleatum* transcribed butyrate synthesis genes to a similar extent both in health and disease; however, in disease both the functional and the taxonomic diversity of expressed butyrate synthesis pathways increased, i.e. butyrate synthesis transcripts from additional taxa and additional enzymatic routes were detected. We then discovered 3 potential functional biomarkers based on the metatranscriptomes which are highly predictive and can discriminate the dysbiotic communities from the healthy ones^27^.

Here we analyzed the data further. We used a newly developed in-house pipeline to gain a deeper understanding of the taxa that drive functional shifts in dysbiosis. Previously, we had used MG-RAST^28^ with the COG database^29^ to characterize the functional alterations of the communities. Here, we used the KEGG database^30^ to assign genes to pathways and functional modules. It provides information not only about the possible general functional category of a gene, but also assigns given genes to specific pathways. By mapping transcripts to KEGG reference genes and using gene set enrichment analysis (GSEA) for pathways or modules, we were able to identify altered pathways or modules as well as those microbial taxa causing them. Additionally, we analyzed archaeal, protozoan and viral transcripts thus unraveling the activity of all three domains of life in periodontitis. Periodontal pocket samples always contain varying amounts of human “contamination” derived from epithelial cells. When we analyzed those transcripts we found hints for possible interactions between the microbiota and the human host.

## Results and Discussion

In this study, we used a dataset from our previous work^27^ which is a part of the “German National Cohort”. These samples were taken from different individuals at several sites (two paper points per site), and the sampling paper points (one paper point per site was chosen) originating from the same individual were pooled together for RNA isolation. Fourteen periodontal pocket metatranscriptome datasets were analyzed, of which 4 were derived from individuals with chronic periodontitis and 10 from periodontally healthy individuals. Study population and methods for sampling, extraction of total RNA, depletion of rRNA, and sequencing have been described^27^. Our previous analyses clearly showed that samples AU_01 and AU_11 are outliers^26, 27^. Those microbial communities were distinct from all other samples for reasons that we do not know. We excluded them from the present analysis because we wanted to identify functional changes in chronic periodontitis occurring consistently in the majority of patients. The short reads aligner BWA with the BWA-MEM^31^ algorithm was utilized to map metatranscriptomic sequences onto DNA sequence references. The following alignment references were utilized: HOMD database^32^ for bacteria, human reference genome (ver. GRCh38), archaeal genomes and viral genomes downloaded from NCBI. For determining *Entamoeba gingivalis* the database of non-redundant 18 rRNA gene sequences provided by SortMeRNA^33^ was used which is based on SILVA^34^. Virulence factors were identified by mapping all putative mRNA reads agains the mvirDB database^35^. Details on numbers of reference genomes, cut-off values and quality controls are provided in the methods or each corresponding result section. The short reads aligner Bowtie2^36^ was employed to eliminate “human contamination” from the metatranscriptome as a preprocessing step for KEGG pathway analysis because Bowtie2 provides a tool for extracting not concordantly mapped read pairs.

The BLASTX like tool DIAMOND^37^ was employed to align non-human putative mRNA reads against KEGG prokaryote peptide sequences since DIAMOND is able to accomplish the mapping for such large amount of sequences within acceptable time and still achieve good enough results comparable to BLASTX.

### Overview - mapping statistics and contribution of bacteria, archaea, protozoa, human and virus transcripts to the metatranscriptome

**Figure 1:**
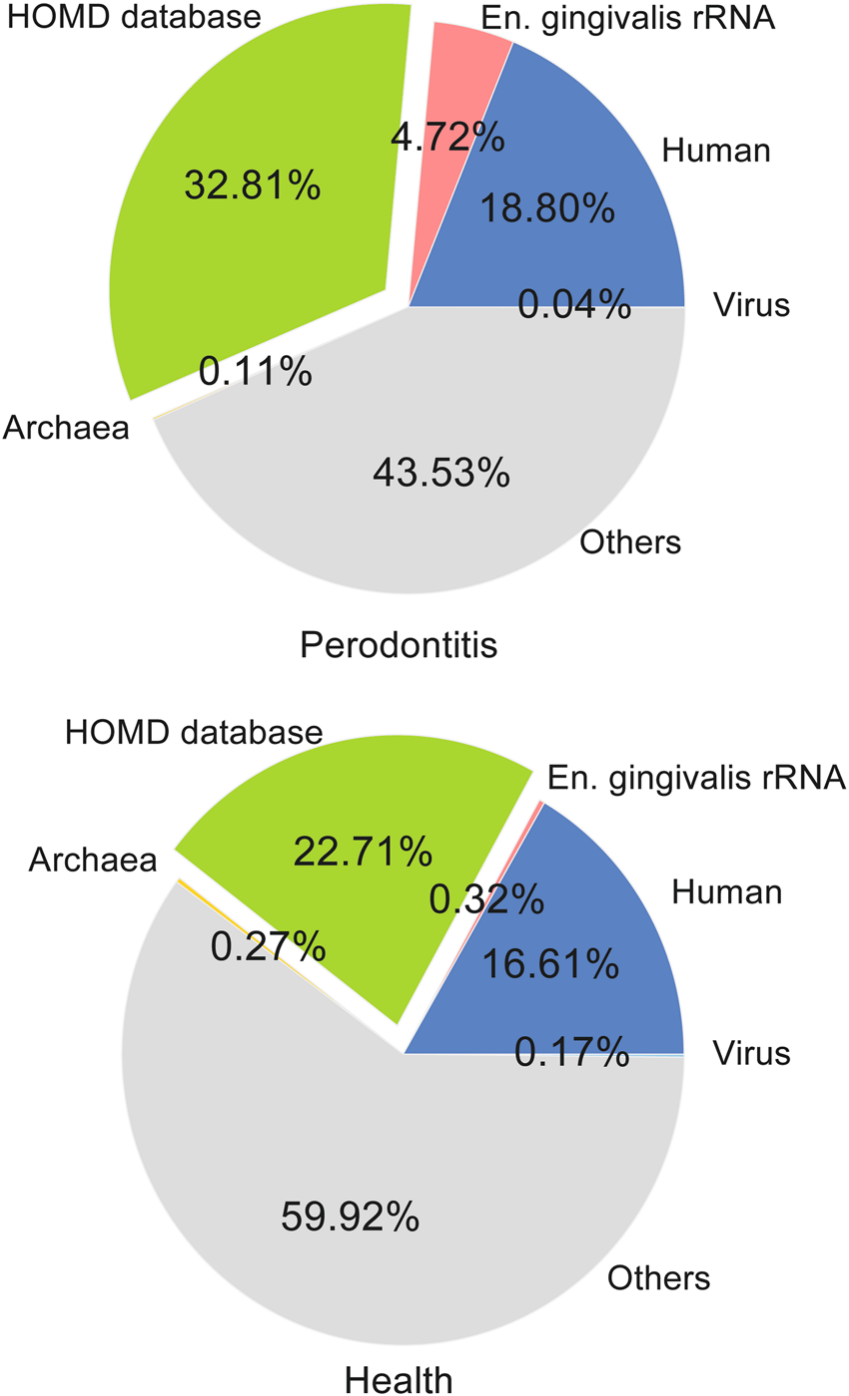
Mapping summary of periodontitis metatranscriptomes. The average proportion of cleaned putative mRNA reads mapped onto HOMD, *En*. *gingivalis* 18S  rRNA (after rRNA removal using SortMeRNA), human reference genome (ver. GRCh38), virus genomes from NCBI database, archaea genomes from NCBI database. The upper panel shows the periodontitis, whereas lower one is the health.

In total, the fourteen samples comprised about 581 million raw reads. After quality control and rRNA removal 59 million reads remained, on average 4.2 million per sample (Supplementary Table 1). Of those reads, 32.8% and 22.7% could be mapped to genomes of bacteria in the HOMD database in periodontitis and health, respectively (Fig. 1, Supplementary Table 1). The contribution of archaea was 0.11% in periodontitis and 0.27% in health. Viruses comprised around 0.04% of all reads in periodontitis and 0.17% in health. The contribution of *Entamoeba gingivalis* transcripts was estimated conservatively based on the 18S rRNA gene sequence, since a fully sequenced genome is not available. Before removal of rRNA by SortMeRNA, *En*. *gingivalis* reads were the second most abundant rRNA found in periodontitis (after human rRNA), accounting for ~6.7 million reads in all samples based on mapping by BWA. This fraction accounted for 6.5% of total RNA reads on average in periodontitis compared with only 0.4% in health, indicating a massive increase in abundance of this protozoon in the disease progression. SortMeRNA removed roughly 90% of rRNA, so even after the rRNA removal there were still 4.7% of all cleaned putative mRNA reads assigned to *En*. *gingivalis* in periodontitis using BWA, whereas this number was 0.3% in health (Fig. 1). BWA and SortMeRNA used different algorithms for alignment. SortMeRNA is designed for fast removal of contaminating rRNA sequences, while BWA is optimized for mapping of short reads.

The proportion of human transcripts was similar in periodontitis and health (18.8% and 16.6%, respectively). The majority of transcripts (43.5% in periodontitis and 59.9% in health) could not be mapped. This reflects a common problem. For mapping, genomes of cultivated bacteria are required, but it is estimated that less than 50% of the species in the oral cavity have been cultivated in the laboratory^38^. In recent years, more and more oral pathogens have been cultivated, described and their genomes have been sequenced, e.g. representatives of Synergistetes and TM7, but this process is slow in relation to the huge diversity of the oral microbiome. Moreover, many microorganisms from the oral cavity rely on co-occurring microbes and thus cannot be cultivated alone. To overcome this bottleneck, deep metagenome sequencing and single cell sequencing methods, which do not rely on cultivation, have been developed^39, 40^.

### Taxonomic composition of bacterial transcripts in health and chronic periodontitis

**Figure 2:**
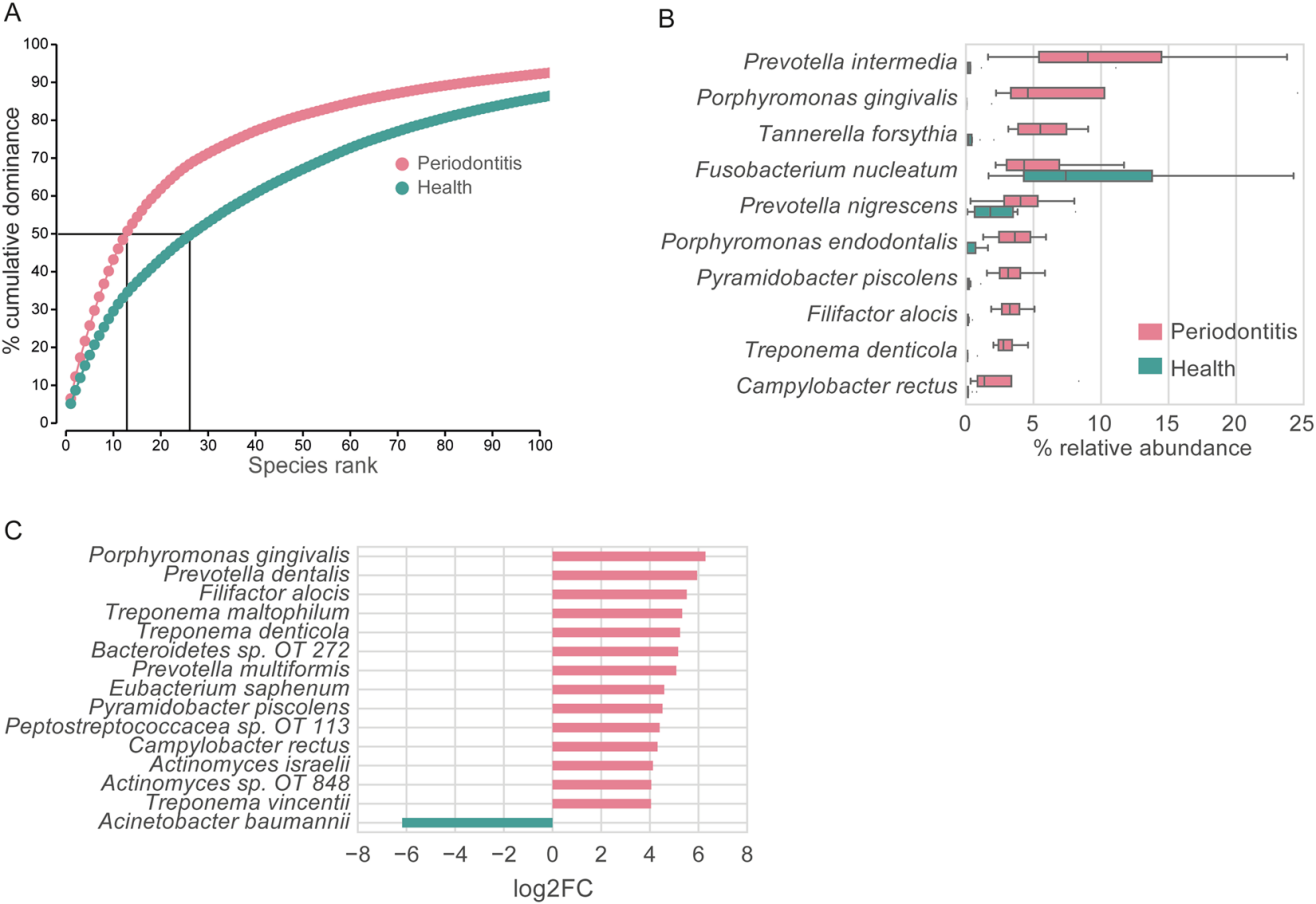
Taxonomic composition of transcripts in health and disease based on HOMD database. (**A**) Dominance plot showing the cumulative average contribution of individual genomes ranked according to transcript abundance of the total mapped reads. (**B**) The relative abundance of transcripts from the top 10 most abundant species in periodontitis in comparison with their abundance in health. (**C**) The differential activity of the microbial species based on the total mapped transcripts. The differential expression analysis was carried out by edgeR. Only species with FDR ≤ 0.05 and log2CPM ≥ 8 and absolute log2FC ≥ 4 are shown. If there were several strains for one species, those transcripts were pooled. Transcripts were mapped to the HOMD database. The bottom and top of the box show the first and third quartiles, the line inside the box indicates the median, and the ends of the whiskers represent the minimum and maximum, the dots on the outside of the whiskers represent outliers.

To understand the shift in the activity of the bacteria at the transcriptional level, we assigned the metatranscriptomes to the HOMD database which contains 460 bacteria genomes using BWA and determined the read counts for each reference genome. For counting the abundance of reads mapped to a specific genome, alignments with mapping quality score (MAPQ) equal to 0 were excluded (20% of mapped reads) because they mapped with similar scores to multiple references. By using PRIMER 7, a dominance analysis based on total transcript abundance per genome was performed. The communities from healthy individuals possessed a much lower dominance level in comparison to dysbiotic communities. The top 13 most abundant genomes in dysbiosis covered more than half of all mapped sequences on average, whereas 27 genomes were required to cover 50% of all mapped reads in health (Fig. 2A, Supplementary Table 2 sheet 1). The data show that the transcriptional activity of the bacteria in the healthy communities is distributed more evenly among species than in dysbiosis, where fewer species dominate the transcriptional profile.

When the genomes were grouped to species, the top 10 most abundant species in periodontitis contributed more than 50% of the transcript reads (Fig. 2B). Among them, *Porphyromonas gingivalis*, *Treponema denticola and Tannerella forsythia* are the so called “red complex” pathogens^41^. Interestingly, *Prevotella intermedia* was the second most dominant bacterium in chronic periodontitis (after *Po*. *gingivalis*). Although *Pr*. *intermedia* is an established oral pathogen belonging to the “orange complex”, the large contribution of its transcripts to the total mapped reads in periodontitis was unexpected^42^. Interestingly, *Prevotella spp*. were found to be predictive for the onset of early childhood caries^43^. Another unexpected finding was the large activity of *Pyramidobacter piscolens* in dysbiosis. *Py*. *piscolens* ^44^ is the first described and genome sequenced member of the Synergistetes phylum isolated from the human oral cavity. Bacteria in this phylum have been found in all habitats; their abundance is increased in periodontal disease, therefore they have been suggested to be opportunistic pathogens^45^.

We then determined the importance of species within the periodontal pockets based not on their abundance, but based on the difference in the total amount of transcripts between health and disease (Supplementary Table 2 sheet 2 and sheet 3). Figure 2C shows that some of the same species are found again (e.g. *Po*. *gingivalis*, *Tr*. *denticola*, *Fi*. *alocis*, *Py*. *piscolens*, *Ca*. *rectus*), indicating that they are not only the most abundant contributors of transcripts in periodontitis, but also those that changed their transcriptional activity most strongly in dysbiosis. Interestingly, *Fu*. *nucleatum*, a potent periodontal pathogen, did not show differential total transcript levels, indicating that it was abundant similarly as in health. Similarly, *Prevotella nigrescens* was found prevalent both in health and periodontitis. Previously we could show that *Pr*. *nigrescens* shifts towards a more virulent phenotype in periodontitis^27^. Finally, several species that contributed only a small percentage to the total metatranscriptome but changed their transcription activity strongly in periodontitis were found. *Eu*. *saphenum* belongs to the “candidate” pathogens for which more detailed investigations are required^46^. Using the stringent cut-offs defined here, transcripts from one species only, namely *Acinetobacter baumannii*, were found highly enriched in health. *Ac*. *baumannii* is an opportunistic pathogen with low virulence which is associated with nosocomial infections and of major concern today because of the emergence of multi-drug resistant isolates^47^. It is unclear why it might be down-regulated in periodontitis relative to health.

### Enrichment of KEGG pathways in periodontitis, identification of key microbial players, and differential expression of virulence factors

The BLASTX like alignment tool DIAMOND was used to map metatranscriptomic reads to KEGG prokaryotic peptide sequences. For annotation of KEGG orthologous (KO) genes only reads were used where both ends had best hits on the same peptide with an identity of the complete read sequence greater than 70% and an e-value smaller than 1e-5. For species-specific gene identification in the KEGG database, sequence identity greater than 90% was required. In total 17.1% of non-human putative mRNA sequences could be annotated with KO genes while 11.3% were annotated with species-specific KEGG genes. The R package edgeR was applied for differential expression analysis for KO genes and KEGG species-specific genes.

**Figure 3:**
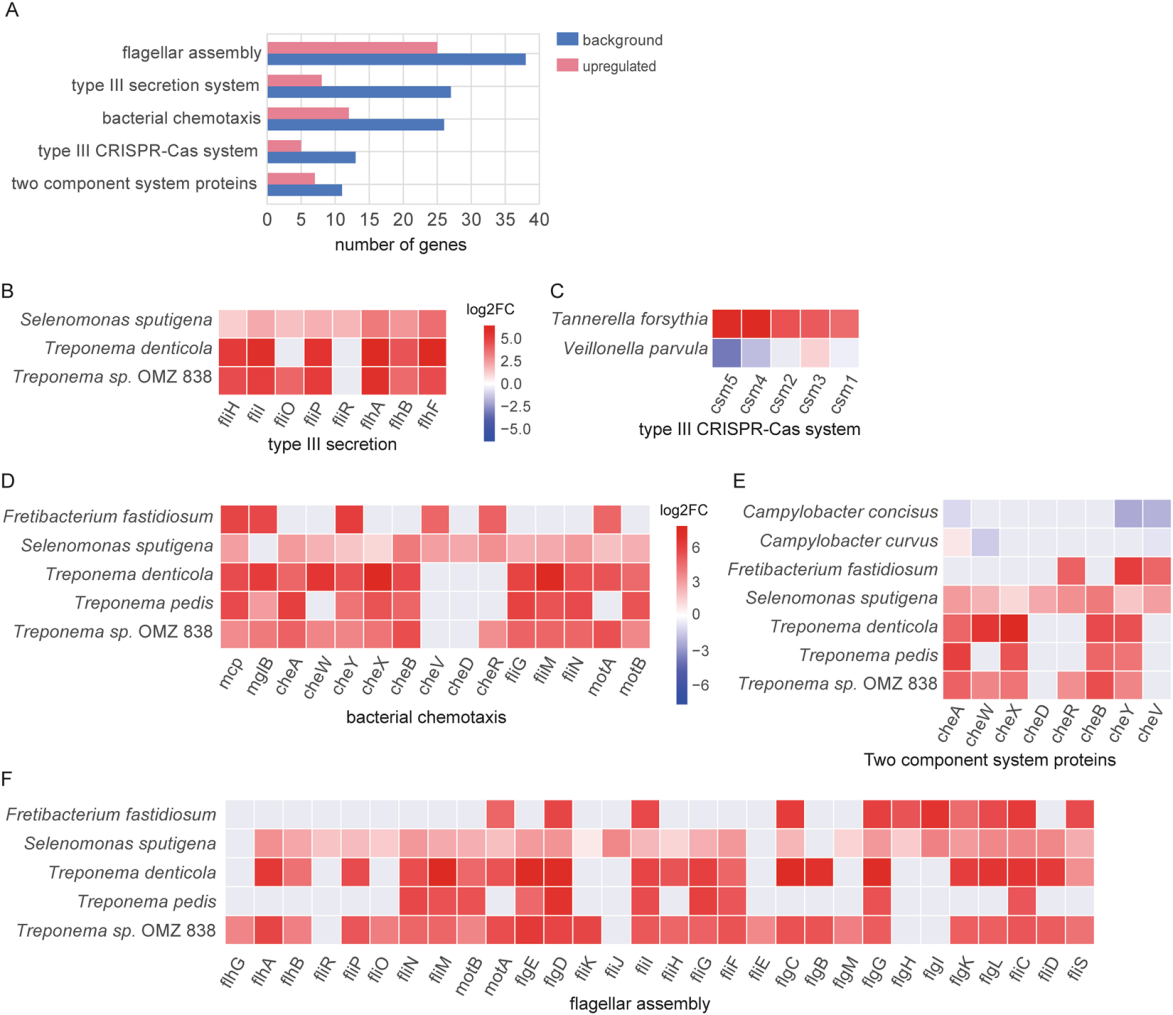
KEGG pathways and functional modules enriched in periodontitis and key microbial players driving the shifts. (**A**) Enrichment of the five most strongly up-regulated KEGG modules in periodontitis. The red bars indicate the number of up-regulated KEGG orthologous gene in the gene set, while dark blue bars show the number of total expressed genes of a given pathway or module. Only the enriched gene sets with FDR < 0.1 and (number of up-regulated genes)/(number of expressed) > 25% are shown here. The complete list of all enriched pathways and modules can be found in Table 1. (**B**–**F**). Key microbial players driving the up-regulation of those functional pathways. Pathways consist of several KO genes (Supplementary Table 3), each of which contains several species specific genes. Only species specific genes with expression level count per million reads (CPM) ≥ 10 are shown. The horizontal axis shows the KEGG orthology genes in the pathway and vertical axis shows the microorganisms in which these KO genes were up- or down-regulated. Note different scales for **B**,**C** and **D**–**F**.

We used GSEA^48^ (Gene Set Enrichment Analysis) to determine enrichment of KEGG pathways and functional modules. The KEGG pathway enrichment analysis was performed based on up-regulated and down-regulated KO genes respectively. Only pathways containing more than 10 expressed KO genes (Supplementary Table 3) were considered. We discovered 18 gene sets which were significantly highly enriched in periodontitis (p < 0.05 and FDR < 0.25, Table 1). Bacterial chemotaxis, flagellar assembly, type III secretion system, type III CRISPR-Cas system and two component system proteins were extremely highly enriched in dysbiotic microbial communities. Figure 3A shows the number of genes up-regulated in periodontitis for these five most strongly enriched modules. These functional modules are highly relevant to virulence and pathogenicity. For example, type III secretion system is a kind of sensory probe protein also called injectisome, which has a needle-like structure helping the bacteria to sense the host cell and inject effector proteins directly into it, thus leading to infection^49^. Flagellin, a component of the flagellum, can modulate pathogenicity and cause a severe host immune response^50^. Using a completely different bioinformatics approach, we had previously identified the flagellar filament core protein FlaB3 from *T*. *denticola* as a highly predictive functional biomarker^27^.

**Table 1:** Enriched KEGG pathways in periodontitis.

Pathway or module	Background^1^	Up^2^	Down^3^	FDR^4^
flagellar assembly	38	25	0	0
rod, hook and filament	19	14	0	0.001
flagellar export apparatus	12	8	0	0.047
bacterial chemotaxis	26	12	0	0.001
type III secretion system	27	8	0	0.001
SEC (secretion) system	12	3	0	0.229
aminotransferase, class III	16	3	0	0.14
arginine and proline metabolism	90	9	0	0.152
alanine, aspartate and glutamate metabolism	44	6	1	0.202
gluconeogenesis, oxaloacetate => fructose-6P	20	5	0	0.162
glycine, serine and threonine metabolism	67	14	1	0.041
nicotinate and nicotinamide metabolism	34	6	0	0.156
type III CRISPR-Cas system	13	5	0	0.089
two component system proteins	11	7	0	0.095
fatty acid biosynthesis, elongation	12	4	0	0.159
butanoate metabolism	69	8	1	0.172
reductive citrate cycle	37	8	1	0.084
one carbon pool by folate	21	5	0	0.149

Gene sets with FDR ≤ 0.25 and number of expressed genes ≥10 are shown. Gene set enrichment was performed as described in Materials and Methods.

^1^Number of expressed KO genes in periodontitis for each enriched pathway.

^2^Number of up-regulated KO genes in periodontitis for each enriched pathway.

^3^Number of down-regulated KO genes in periodontitis for each enriched pathway.

^4^FDR of statistical test for the gene set emrichment analysis.

For gaining a deeper understanding of the pathogenesis of periodontitis, it is crucial to determine which species contribute to the functional shifts in the dysbiotic community. We performed a differential expression (DE) analysis between health and periodontitis based on the expression level of KEGG species-specific genes, which revealed that the upregulation of these enriched functions was mainly driven by several key microbial players (Fig. 3B–F). Three species of Treponema (*Tr*. *denticola*, *Tr*. *pedis*, *Tr*. *sp*. OMZ 838) contributed to the upregulation of four enriched modules, namely type III secretion systems, chemotaxis, flagellar assembly and two component system proteins and thus were the key species for those functions. *Ta*. *forsythia* was the only species that contributed strongly to the enrichment of the type III CRISPR-Cas system. *Fr*. *fastidiosum* and *Se*. *sputigena* contributed to the upregulation of bacterial chemotaxis, flagellar assembly and two component system proteins. They are poorly characterized bacteria but since they have frequently been identified in culture-independent studies, their prevalence in periodontal disease was systematically investigated and shown to be significant^46^. *Fr*. *fastidiosum* is the first cultivated representative of a lineage belonging to the phylum Synergistetes; interestingly, it grows only in co-culture or with culture extracts of other oral bacteria^51^. *Se*. *sputigena* produces lipopolysaccharide (LPS) which is a major factor in the pathogenesis of periodontal diseases^52^.

We also observed, although with an FDR value higher than 0.25, that some important virulence related functional modules, namely LPS biosynthesis (enrichment p = 0.160, FDR = 0.462), type II toxin-antitoxin system (TA system, enrichment p = 0.058, FDR = 0.264), iron uptake and acquisition proteins were highly expressed in active key microbial players (Supplementary Fig. 2, species specific genes with log2CPM ≥ 4.5 and p ≤ 0.05 for DE analysis are shown), e.g. *Po gingivalis*, *Pr*. *Intermedia*, *Fr*. *fastidiosum*, *Tr*. *denticola*, *Ta*. *forsythia*, and *Fi*. *alocis*, which are considered as periodontal pathogens. LPS is a virulence factor of Gram-negative microorganisms which can impair the immune response of the host^53^. TA systems are required for the maintenance of plasmids or genomic islands^54^ and their upregulation can enhance the stress tolerance and drug resistance of the bacteria^55^. Iron plays an important role in basic biological processes in bacteria and is essential for the pathogenesis of pathogens^56^. *Po*. *gingivalis* and *Pr*. *intermedia* were the main contributors to all three modules, confirming their importance for chronic periodontitis.

Mapping the reads of the metatranscriptomes to the mvirDB database^35^ using BWA showed that 9 virulence factors (Table 2) were strikingly differentially expressed in periodontitis with log2FC values above 9, indicating that those transcripts were 500 times more abundant in dysbiosis. Because of the strong enrichment of *Po*. *gingivalis* in periodontitis, most of them are virulence factors of this major oral pathogen. Fimbrillin is the major subunit of fimbriae of *Po*. *gingivalis* which mediates the adhesion and colonization of the host cell^57^. The immunoreactive 47 kD antigen epsC of *Po*. *gingivalis* (also called UDP-N-acetylglucosamine 2-epimerase) is a LPS biosynthesis protein which can arouse a strong host immune response. Dentilisin is a major surface protease of *Tr*. *denticola* which can hydrolyze host proteins and inflammatory cytokines^58^.

**Table 2:** Differentially expressed virulence factors in health and periodontitis.

Virulence factors	log2FC	log2CPM	Organism
fimA type 1b	10.98	13.09	*Po*. *gingivalis*
fimA type 1	11.58	12.47	*Po*. *gingivalis*
Immunoreactive 47 kD antigen	10.42	11.34	*Po*. *gingivalis*
Hemagglutinin A (hagA)	8.52	16.76	*Po*. *gingivalis*
Arginine deiminase (arcA)	7.94	12.88	*Po*. *gingivalis*
Serine protease dentilisin	9.07	11.71	*Tr*. *denticola*
Flagellar motor switch protein (FliG)	7.12	9.56	*Tr*. *denticola*
fimA type 3	9.05	12.19	*Po*. *gingivalis*
Arginine deiminase (arcA)	7.07	9.11	*My*. *arginini*

Reads were mapped against MvirDB to investigate the expression level of virulence factors. The table lists the differentially expressed virulence factor genes calculated by edgeR.

The peptidyl-arginine deiminase (PAD) is another important virulence factor of *Po*. *gingivalis* ^59^. PAD performs a post-translational modification of proteins by converting terminal arginine residues into a non-coded amino acid, citrulline, which leads to conformational and functional changes of the protein and yields NH_3_, hence the importance of this enzyme for acid adaptation^60^. The PAD produced by *Po*. *gingivalis* (PPAD) belongs to a new super-family of PAD enzymes (pfam04371) distinct from the human protein (https://www.ncbi.nlm.nih.gov/Structure/cdd/cddsrv.cgi?uid=282257). It is able to modify free arginine as well as arginine within human proteins and peptides^61^. Since citrullinated proteins are strong antigens and thus trigger autoimmunity, this mechanism could be the causal link between periodontitis and autoimmunity related diseases such as rheumatoid arthritis^61^. Supporting this hypothesis, we found 250 fold overexpression of PPAD in periodontitis. The transcript was not found in other species of *Porphyromonas* which were also enriched in periodontitis, e.g. *Po*. *endodontalis* (Fig. 2), in accordance with a recent study confirming that this enzyme is unique to *Po*. *gingivalis* ^62^. Interestingly, we discovered a highly differentially expressed transcript of PAD belonging to *My*. *arginini*. This protein has 24% identity with PPAD. Thus, it remains to be tested if it is able to citrullinate free arginine and/or human proteins in a similar way as PPAD. *My*. *arginini* transcripts had a low prevalence in all samples except in one dysbiotic community (AU_13). Mycoplasma is a genus of bacteria lacking a cell wall. The bacteria from this genus, e.g. *My*. *arginini*, *My*. *arthritidis*, and *My*. *neurolyticum* can modulate the host immune response and are involved in a wide variety of immune diseases^63, 64^. It would be very interesting to study the function of PAD of *My*. *arginini* to determine if it is the second known oral pathogen able to trigger autoimmunity by citrullination.

### Virus transcripts in the periodontal microbiota

**Figure 4:**
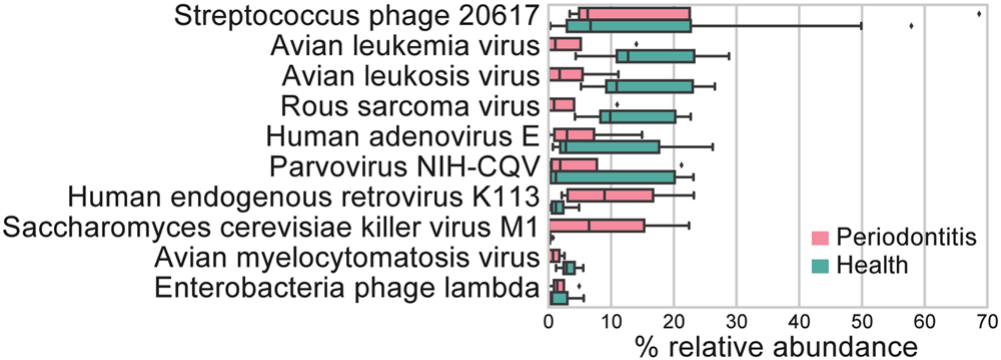
Virus transcripts in periodontal pockets. All virus genomes from NCBI database were used as mapping reference. Ten most abundant viruses are shown which accounted for about 80% of viral sequences. The bottom and top of the box show the first and third quartiles, the line inside the box indicates the median, and the ends of the whiskers represent the minimum and maximum, the dots on the outside of the whiskers represent outliers.

The contribution of viruses was determined by mapping the putative mRNA reads onto 4418 virus genomes from the NCBI database using BWA. In total, viruses contributed 0.13% of total putative mRNA reads. Since they have extremely small genomes compared to bacteria, their number in the community was enormous. BWA discovered 10 species which accounted for about 80% of viral transcripts. The mapped reads with MAPQ = 0 were filtered out before counting the abundance of different viruses. Streptococcus phage was the most abundant virus both in health and periodontitis which constituted about 20% of virus sequences on average (Fig. 4, Supplementary Table 4). Human endogenous retrovirus K113 was abundant in periodontitis but rare in health. Parvovirus NIH-CQV has previously been shown to be a contamination from the RNA extraction kits^65^ and was identified at very low abundance in this study.

### Transcriptional activity of archaea in periodontal microbiota

**Figure 5:**
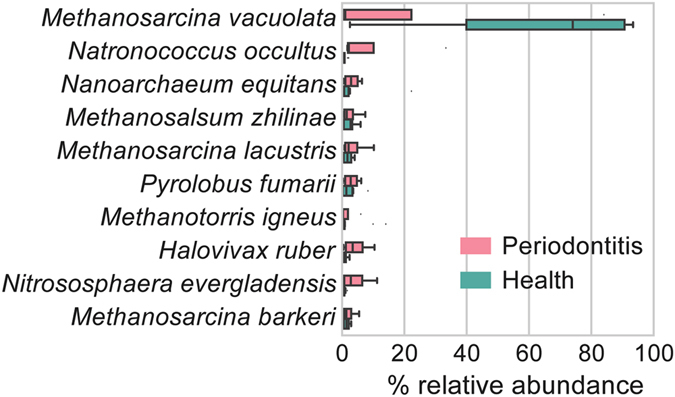
Transcriptional activity of archaea in the periodontal pocket microbiota. All archaea genomes from NCBI database were used as mapping reference. Top 10 most abundant archaea species across all subjects accounted for 72% of total archaeal reads. The bottom and top of the box show the first and third quartiles, the line inside the box indicates the median, and the ends of the whiskers represent the minimum and maximum, the dots on the outside of the whiskers represent outliers.

The reads were mapped against 204 archaea genomes downloaded from NCBI using BWA to determine the transcriptional abundance of archaea in the communities. HOMD genome reference presently contains only one archaea genome. Transcripts of archaea accounted for 0.22% of total putative mRNA reads in the metatranscriptome. Only reads mapped onto the reference with MAPQ higher than 0 were considered as valid alignment for species detection. The 10 most abundant archaea accounted for more than 72% of archaea reads on average. *Methanosarcina vacuolata* was the most abundant archaeal species both in periodontitis and health accounting for 21.5 and 62.5 percent of total archaeal sequences, respectively (Fig. 5, Supplementary Table 5). *Me*. *vacuolata* is an anaerobic methanogenic archaea species which is poorly described. Five out of these 10 archaea are methanogens. Interestingly, we found methanogens to be more abundant in healthy subjects. The methanogen *Me*. *mazei* was frequently detected in the oral microbiome previously^15^ but it was a minor constituent of the archaeal community in terms of the total transcriptional activity in this study.

### *Entamoeba gingivalis* in dysbiotic periodontal communities

**Figure 6:**
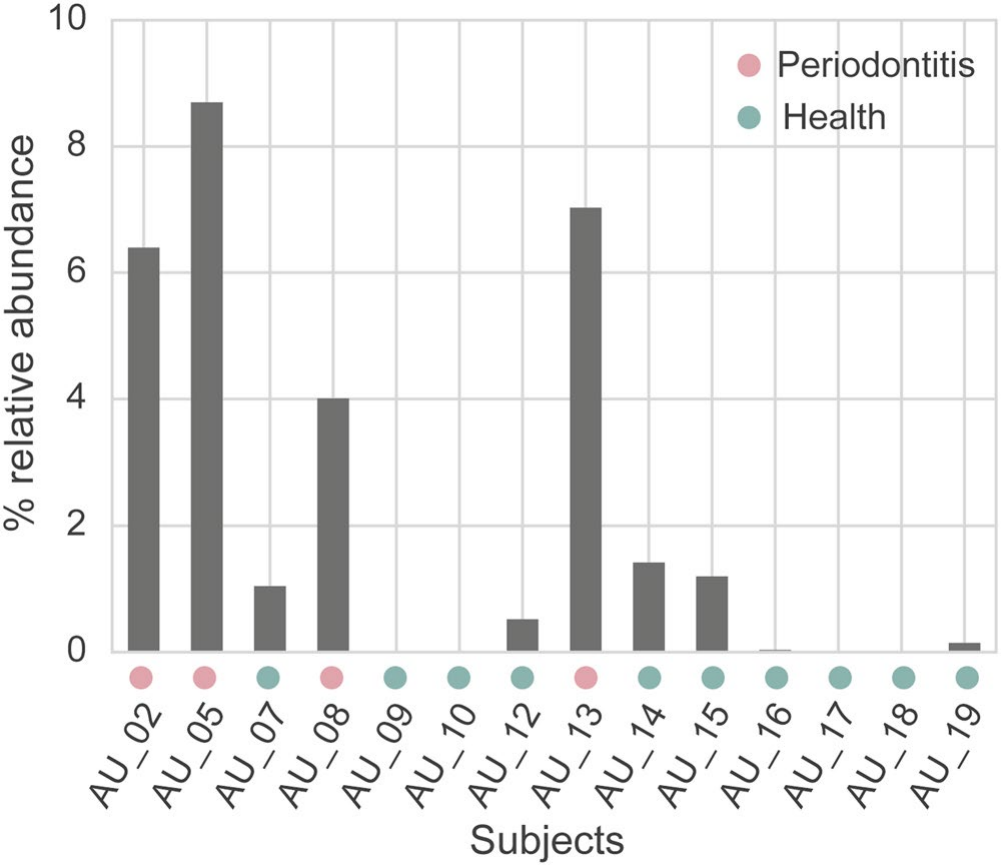
Abundant *En*. *gingivalis* in periodontitis. Total reads (without rRNA removal using SortMeRNA) from each sample were mapped against the 18S rRNA gene of *En*. *gingivalis*.

*Entamoeba gingivalis* is a parasitic protozoan usually found in periodontal pockets from patients with periodontitis but seldom from persons with a healthy gum^25^. To investigate its presence we mapped total RNA reads (before *in silico* removing rRNA) using BWA against the non-redundant 18S rRNA gene database provided by SortMeRNA which contains the 18S rRNA gene of *En*. *gingivalis*as well as two other *Entamoeba* and is based on SILVA. Among the reads mapped to the 18S rRNA gene of *En*. *gingivalis*, more than 72% had a sequence similarity higher than 99% (calculated based on the edit distance of the alignments reported by BWA). Figure 6 shows that the 18S rRNA sequence of *En*. *gingivalis* was extremely abundant (up to 9% of all reads) in all four subjects with periodontitis but rare (up to 2%) or absent in healthy individuals, confirming previous studies. The precise role of *En*. *gingivalis* in periodontitis is hard to elucidate because its genome is still lacking.

### The response of the human host

Human transcripts accounted for 18.8% and 16.6% of putative mRNA reads in periodontitis and health, respectively. We identified 14 human genes which were differentially expressed (Table 3). All of them were up-regulated in periodontitis. The upregulation of the ferritin light chain (ferric iron binding protein) is intriguing, since we discovered that many microbial genes involved in iron acquisition were also up-regulated in the dysbiotic community. This implies that there is a strong competition between bacteria and host for iron. Four up-regulated human genes in periodontitis belonged to nucleolar phosphoprotein B23 (NPM1). Their overall abundance was low (log2CPM around 4), but they were strongly (up to log2FC 6.15) overexpressed in periodontitis. Overexpression of this protein has been proven to be associated to various types of cancer of diverse histological origins including oral cancer^66^. NPM1 is considered as a tumor marker and a putative proto-oncogene^67^. It is believed that NPM1 promotes tumor growth by inactivating the tumor suppressor p53/ARF pathway. Low levels of NPM1 were shown to suppress tumor growth by inhibiting the centrosome amplification^67^.

**Table 3:** Differentially expressed human genes between health and periodontitis in periodontal pockets.

Gene symbol	log2FC	log2CPM	Annotation
ANKRD30BL	9.07	11.31	ankyrin repeat domain 30B-like
NPM1P35	5.49	4.94	nucleophosmin 1 (nucleolar phosphoprotein B23, numatrin) pseudogene 35
LOC105374556	5.04	4.78	uncharacterized LOC105374556
ATAD2	3.81	6.71	ATPase family AAA domain-containing protein 2 (EC 3.6.1.3) (AAA nuclear coregulator cancer-associated protein) (ANCCA)
NPM1P4	6.15	3.51	nucleophosmin 1 (nucleolar phosphoprotein B23, numatrin) pseudogene 4
LOC101930010	5.62	6.28	uncharacterized LOC101930010
ENO3	4.42	3.50	Beta-enolase (EC 4.2.1.11) (2-phospho-D-glycerate hydro-lyase) (Enolase 3) (Muscle-specific enolase) (MSE) (Skeletal muscle enolase)
MT-TP	4.62	4.45	mitochondrially encoded tRNA proline
RNA18SP2	6.24	3.71	RNA, 18S ribosomal pseudogene 2
NPM1P31	4.16	4.12	nucleophosmin 1 (nucleolar phosphoprotein B23, numatrin) pseudogene 31
NPM1P24	5.52	4.00	nucleophosmin 1 (nucleolar phosphoprotein B23, numatrin) pseudogene 24
SSR4	4.31	3.08	Translocon-associated protein subunit delta (TRAP-delta) (Signal sequence receptor subunit delta) (SSR-delta)
HMGB1P10	4.28	4.46	high mobility group box 1 pseudogene 10
FTL	2.38	7.30	Ferritin light chain (Ferritin L subunit)

Reads were mapped against the human reference genome (ver. GRCh38) to investigate the response of the host. The table lists the differentially expressed host genes calculated by edgeR.

The most strongly up- regulated and most abundant gene (log2FC 9.1, log2CPM 11.31) among all differentially expressed human genes was the ankyrin repeat domain 30B-like gene (ANKRD30BL). Ankyrin repeat domain is a 33-residue motif in proteins which plays an important role in protein-protein interactions^68^. Overexpression and mutation of ankyrin repeats is associated with various human diseases including cancer, cardiovascular disease and neurological disorder^69, 70^. LOC101930010 is a validated but uncharacterized long non-coding RNA, and LOC105374556 is an unknown non-coding RNA. Finally, a beta-enolase gene was up-regulated. This protein is involved in energy metabolism in cancer cells^71^. These findings support the hypothesis that chronic infections like chronic periodontitis are associated with an increased risk for cancer development.

## Conclusion

Here we have investigated the transcriptional activity of organisms from all three domains of life in periodontal pockets. From these data, a number of hypotheses regarding dysbiosis can be developed.

Bacterial communities from healthy individuals had a higher taxonomic and functional diversity. In dysbiosis, several key players gave rise to the upregulation of virulence related functional modules, e.g. two component system proteins, flagellar system, CRISPR-Cas system, type III secretion system, and bacterial chemotaxis. The active key drivers in the dysbiotic communities were the red-complex pathogens, e.g. *Po*. *gingivalis*, *Tr*. *denticola*, and *Ta*. *forsythia*. However, we also found strong evidence for the importance of candidate pathogens like *Pr*. *intermedia*, *Fr*. *fastidiosum*, *Se*. *sputigena*, and *Fi*. *alocis* in periodontitis.

Many virulence factors were enriched in dysbiosis, including the PAD enzyme of *Po*. *gingivalis* (PPAD), with is thought to be causative for the strong autoimmune response in periodontitis and its epidemiological link to rheumatoid arthritis^61^. Interestingly, we discovered expression of a PAD gene from *My*. *arginini* which was highly differentially transcribed in chronic periodontitis and which encodes a protein with 24% sequence similarity to PPAD. If it could be experimentally confirmed that this enzyme is able to citrullinate human proteins this would be the first such enzyme discovered outside of *Po*. *gingivalis* thus providing a second route for triggering autoimmune responses in dysbiosis.

We could confirm the importance of methanogens as the most abundant archaea in periodontal pockets, but we found them to be more abundant in health and to be dominated by massively understudied species.

This is the first study investigating gene expression from human tissue in periodontal pockets together with that of the microbiota. We hypothesize that competition for iron occurs in inflamed periodontal tissue, since a ferritin gene was up-regulated in the host tissue while various iron uptake related genes were up-regulated in key microbial players in the dysbiotic communities. It is similar in other infections, e.g. *Pseudomonas aeruginosa* in cystic fibrosis^72^.

Remarkably, the majority of the human genes up-regulated in periodontitis were identified as tumor markers or have been shown to play a role in cancer development. These findings have to be interpreted with caution, because they rely on 14, samples only, from which 4 where fr﻿om individuals with chronic periodontitis. Yet since they were highly significant, and since the chronic periodontitis patients have been carrying the inflammation for a prolonged time in their life, the transcripts expressed in the human tissue may reflect a systemic response. The data support the hypothesis that periodontitis can increase the risk of cancer development. Since we have a relatively small sample size in this study further validation and investigation is required to provide compelling evidence and to generalize our findings to population level.

## Materials and Methods

### Bioinformatics pipeline and preprocessing of sequencing data

The pipeline for data processing and analysis is illustrated in Fig. S3. Primers and sequencing adaptors were removed from raw sequencing data, followed by clipping the low quality score bases from the reads to achieve cleaned reads with Fastq-Mcf^73^. Thereafter, the rRNA reads were eliminated by using SortMeRNA v2.0.

### Short reads alignment

For determining the dominant species and identifying those microorganisms most differentially active between dysbiotic and healthy communities, we used the collection of reference genomes for the HOMD database as genome reference database containing 460 bacterial genomes and BWA as the aligner. During reads mapping using BWA, the BWA-MEM algorithm along with mapping seed length of 31which is much longer than the default seed length 19 was applied to achieve more reliable alignments. The default Smith-Waterman scoring strategy of BWA-MEM was applied in which alignments with Smith-Waterman score higher than 30 were reported.

To avoid a biased counting for the transcript abundance of species, the reads mapped with MAPQ equal to 0 were excluded (the same when counting for archaea and virus species).

Expression of the viruses and archaea was investigated by mapping the cleaned putative mRNA reads against all complete virus and archaea genomes available from NCBI using BWA. The MvirDB database consisting of 28,966 virulence factor genes was chosen as the reference for virulence factors, and BWA was used as aligner.

To investigate the presence of *En*. *gingivalis*, all quality controlled RNA reads without removing rRNA sequences *in silico* were mapped against the 7348 non redundant 18S rRNA genes from SILVA using BWA.

Human transcripts were detected by mapping the reads against the human genome (ver. GRCh38) from the NCBI database using BWA. The expression level of human genes was calculated using FeatureCounts^74^.

### Differential expression analysis

All DE analyses were computed by the R package edgeR with exact test and p-values were corrected with method “Benjamini-Hochberg” for multiple comparison. A corrected p-value FDR <= 0.05 was considered as significant in DE analysis.

### KEGG pathway enrichment analysis

For KEGG pathway enrichment analysis, we utilized the protein sequences of the KEGG database as an alignment reference (Fig. S3C). It represents a non-redundant dataset on the prokaryote (bacteria and archaea) species level and contains about 7 million non-redundant peptide sequences grouped into 14,390 distinct KEGG orthologous (KO) genes. Each sequence has a KEGG gene identifier linking to one or several KO gene numbers (KO identifier or K number). DIAMOND, a much faster alternative to BLASTX was employed to accomplish the sequence alignment. Before mapping, human reads were excluded using Bowtie2 by extracting reads that did not align concordantly to the human genome. Only reads having both ends assigned to the same gene as the best hit with sequence identity >= 70 and e-value <= 1e-5 were taken into account. The KEGG orthologous gene numbers (KO identifier or K number) were thereafter assigned to each corresponding gene using an in-house Python script based on the gene_KO id-mapping file from the KEGG database. For retrieving the differential expression (DE) of all genes for each species, we adopted the same sequence alignment reference (KEGG prokaryote proteins) but more strict criteria for counting, namely identity >= 90, e-value <= 1e-5, and both ends had to align to the same best hit protein sequence.

GSEA^48^ was used for KEGG pathway enrichment calculation. In GSEA, the significance of enrichment is tested based on the Kolmogorov-Smirnov statistical test. The FDR cutoff 0.25 is suggested by the GSEA manual for relatively small number of gene sets being analyzed. According to the instructions of GSEA for RNA-seq data analysis, we first performed a DE analysis using edgeR^75^ to generate a pre-ranked gene list as input for GSEA in terms of the log2FC and FDR. After completion of calculation, output gene sets with less than 10 expressed genes or with p greater than 0.05 and FDR above 0.25 were excluded from the result.

### Statistics

“Benjamini-Hochberg” method was used to correct p-value for multiple comparison. All boxplots presented in this study delineate the minimum, first quartile, median, third quartile and outliers of the data. The bottom and top of the box show the first and third quartiles, the line inside the box indicates the median, and the ends of the whiskers represent the minimum and maximum, the dots on the outside of the whiskers represent outliers.

## Electronic supplementary material

Supplementary Information

Dataset S1

Dataset S2

Dataset S3

Dataset S4

Dataset S3
