# Supplementary material for: Dysbiosis in chronic periodontitis: Key microbial players and interactions with the human host
Source: Sci Rep. 2017 Jun 16;7:3703. doi: 10.1038/s41598-017-03804-8 (PMC5473847; doi:10.1038/s41598-017-03804-8)
Supplement: Supplementary file 1 — Supplementary Information [file 41598_2017_3804_MOESM1_ESM.pdf]

Dysbiosis in chronic periodontitis: Key microbial players and interactions with the human host

Zhi-Luo Deng[1], Szymon P. Szafrański[1], Michael Jarek[2], Sabin Bhuju[2], and Irene Wagner-Döbler[1*]

[1]Research Group Microbial Communication, Department of Molecular Infection Biology, Helmholtz-Centre for Infection Research (HZI), Braunschweig, Germany

[2]Genome Analytics, Helmholtz Centre for Infection Research, Braunschweig, Germany

[*]Corresponding author: Irene.Wagner-Doebler@helmholtz-hzi.de

Supplementary information

Figure S1. Clustering of the periodontal communities based on the abundance of total transcripts from the respective genomes from HOMD database. The Bray-Curtis was used for calculating the similarities between samples.

Figure S2. The microbial players contributing to the shifts of LPS biosynthesis, type II TA system, iron transport system. Only differentially expressed species specific genes with log2CPM ≥ 4.5 and $p \leq 0.05$ are shown.

Figure S3. The bioinformatics pipeline. (A) Quality control and removal of primers and rRNA reads (B) Data processing (C) Enrichment analysis of KEGG pathways and functional modules.

Table S1. The mapping rate summary for the metatranscriptomes of each sample. The details of the mapping are described in Materials and methods.

Table S2. The composition of bacteria based on transcripts, HOMD was used as reference database

(sheet 1). The composition (% relative abundance) of bacteria based on transcript reads mapped onto all reference genomes

(sheet 2). The composition (% relative abundance) of bacterial species (genomes were grouped into species)

(Sheet 3). Differential expression analysis based on the total transcriptional abundance of each species

Table S3. KEGG enrichment analysis

(sheet 1). Differential expression analysis for KO genes

(sheet 2). KEGG Ortholog (KO) identifier, abbreviation for the KO genes and functional annotation used in this study

(sheet 3). Differential expression analysis for species specific genes

(sheet 4). Gene set of five extremely enriched KEGG pathways and modules

Table S4. The relative abundance (%) of viruses based on all reads mapped onto NCBI viral reference genomes

Table S5. The relative abundance (%) of archaea based on all reads mapped onto NCBI archaea reference genomes

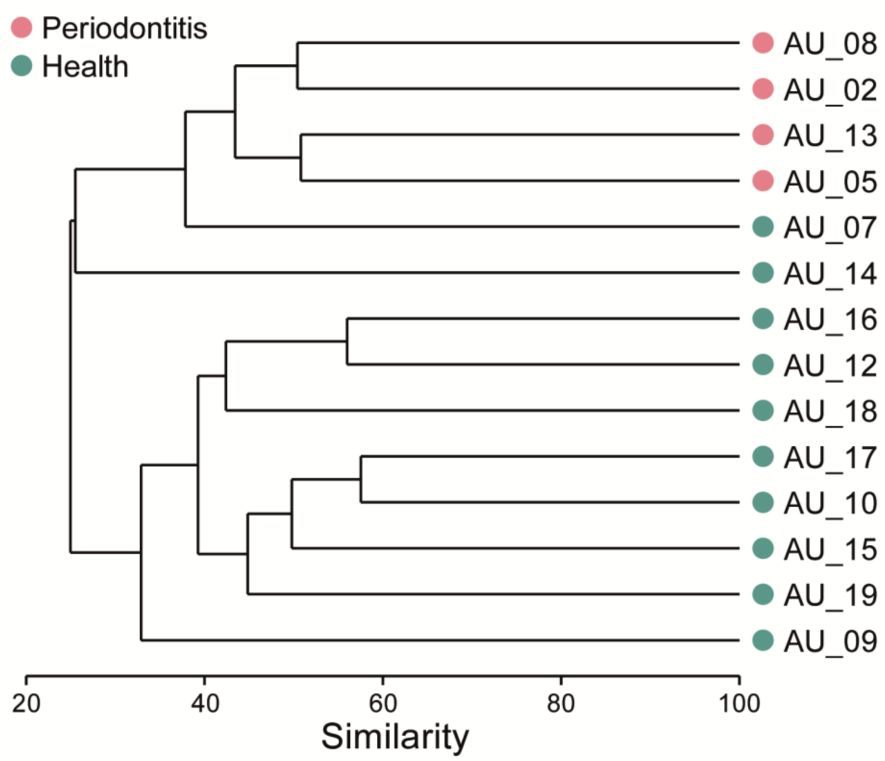

Figure S1. Clustering of the periodontal communities based on the abundance of total transcripts from the respective genomes from HOMD database

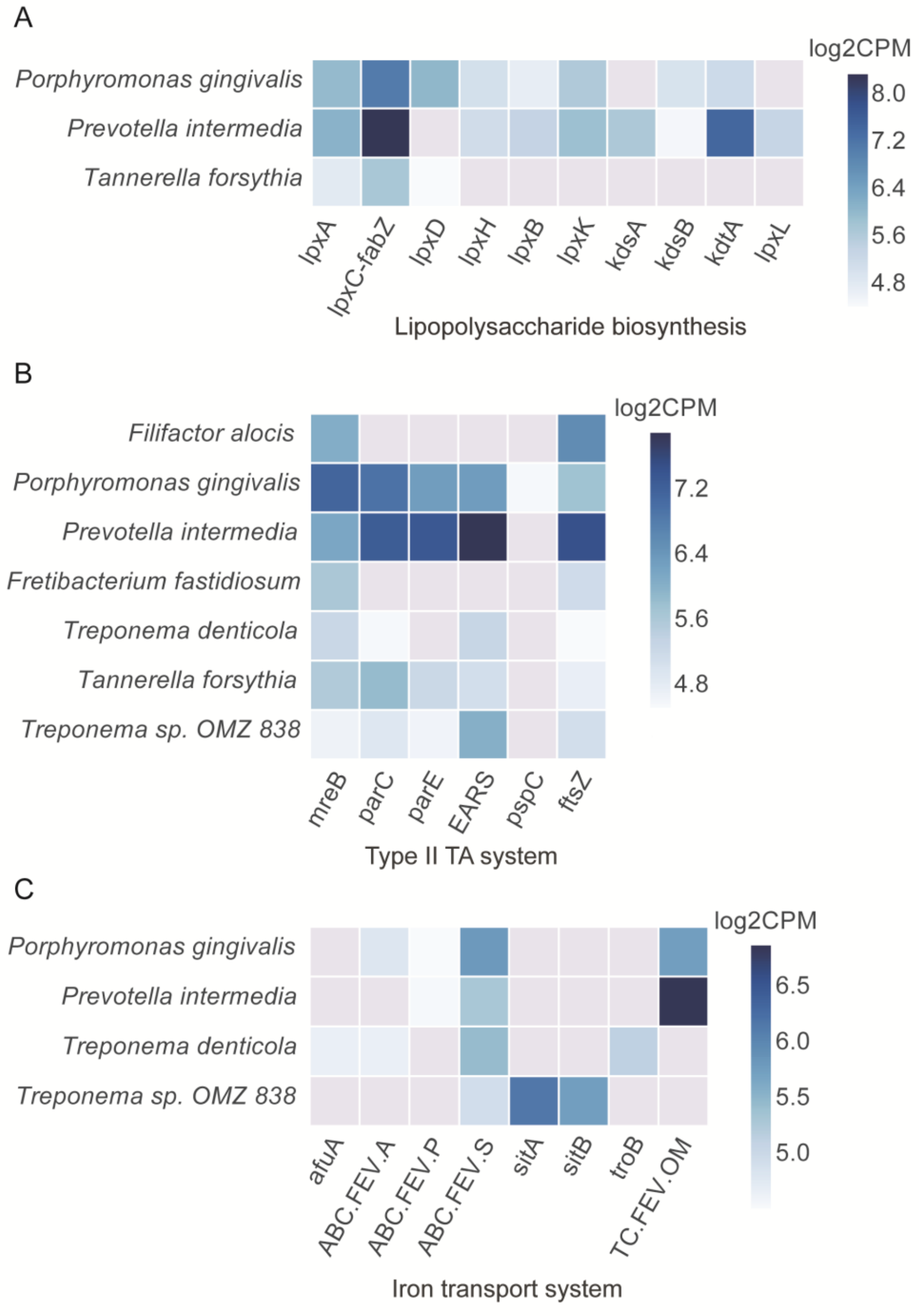

Figure S2. The microbial players contributing to the shifts of LPS biosynthesis, type II TA system, iron transport system

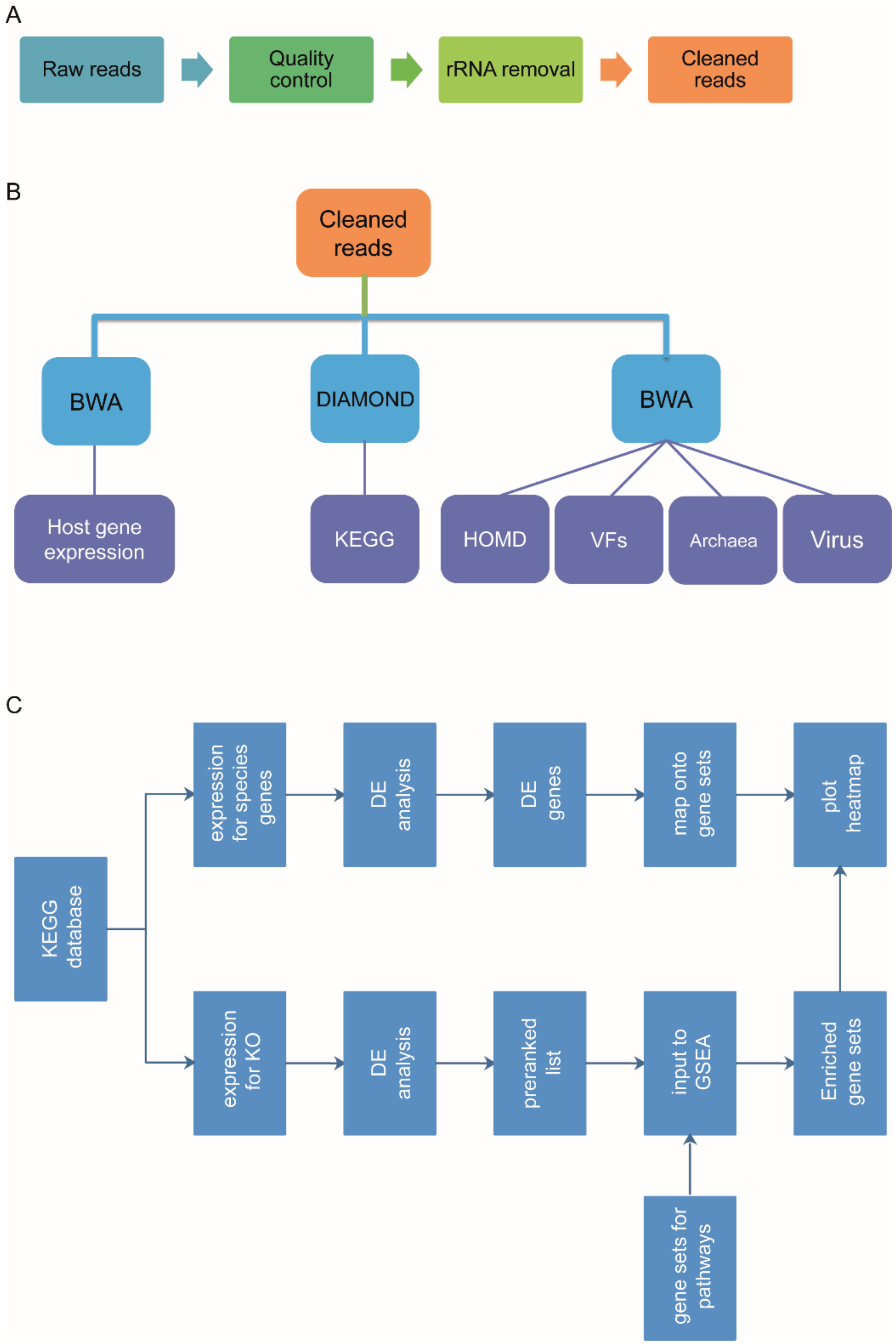

Figure S3. The bioinformatics pipeline
